# Supplementary material for: O-acyltransferase genes involved in the production of volatile sex pheromones in Caenorhabditis elegans
Source: Proc Natl Acad Sci U S A. 2026 Jan 7;123(2):e2524778123. doi: 10.1073/pnas.2524778123 (PMC12799122; doi:10.1073/pnas.2524778123)
Supplement: Supplementary file 1 — Appendix 01 (PDF) [file pnas.2524778123.sapp.pdf]

PNAS

O-Acyltransferase Genes Involved in the Production of Volatile Sex Pheromones in *Caenorhabditis elegans*

Xuan Wan[1], Sarah M Cohen[1], Yan Yu[2], Henry Hoan Le[2], Heenam Park[1], Alessandro Groaz[1], Rachel Moreno[3], Minyi Tan[1], Jessica Schneider[1], Matthew R. Gronquist[4], Ryoji Shinya[5], Frank C. Schroeder[2], and Paul W Sternberg[1]*

Paul W. Sternberg
Email: pws@caltech.edu

This PDF file includes:

Figures S1 to S7
Tables S1 to S4

Supporting Information Text

Supplementary Results.

Additional results and supporting analyses are presented here and in Figs. S1–S7, including full chromatograms and mass spectra for pheromone-positive and pheromone-negative samples, brood-size and somatic growth trajectories for *oac* mutants, comparative analyses of O-acyltransferase family members across nematode species, and phylogenetic and sequence analyses of *oac* family genes.

Supplementary Method.

Additional information of CRISPR generation of *oac* alleles. CRISPR–Cas9 genome editing was used to generate a comprehensive panel of *oac* alleles, including precise deletions and triple-stop insertion alleles, using sgRNAs targeting conserved exons and repair templates providing 35–60 bp homology arms. Table S1 summarizes all CRISPR-generated oac alleles used in this study, including gene ID, allele type, strain name, and a brief description of the engineered lesion. Tables S2 and S3 provide the full molecular details for deletion and insertion alleles, respectively, listing for each strain the left and right homology arm sequences and the forward and reverse genotyping primers. Table S4 compiles a master list of all *oac* mutant strains used in the work, indicating for each genotype the strain designation and source.

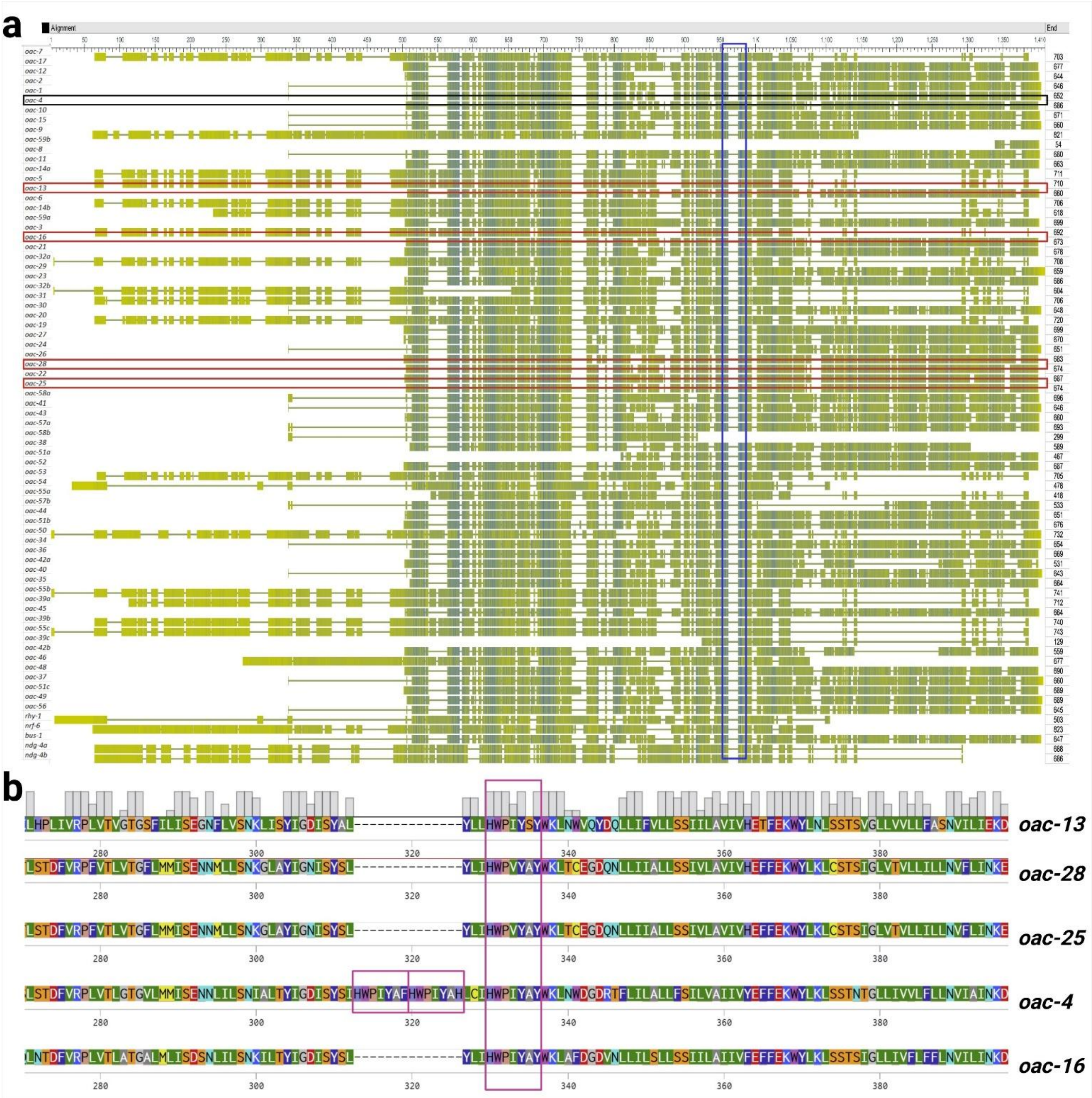

Fig. S1. Conserved SYH catalytic motif in OAC acyltransferase 3 domains. (a) Sequence alignment of OAC proteins with acyltransferase 3 domains, visualized using BLOSUM substitution matrices. Residue conservation is color-coded: blue = high similarity, green = low similarity. Red boxes highlight *oac* family genes linked to VSPs synthesis in this study; black box denotes *oac-4*. The SYH catalytic motif (blue box) is conserved across all OAC family members. (b) The protein sequence of the *oac-4* insertion sequence within the SYH catalytic motif. *oac-4* uniquely contains two HWPIYA[F/H] fragment insertions (pink boxes) within this motif. Multiple sequence alignment of OAC proteins involved in VSP synthesis and the evolutionary related gene *oac-4*.

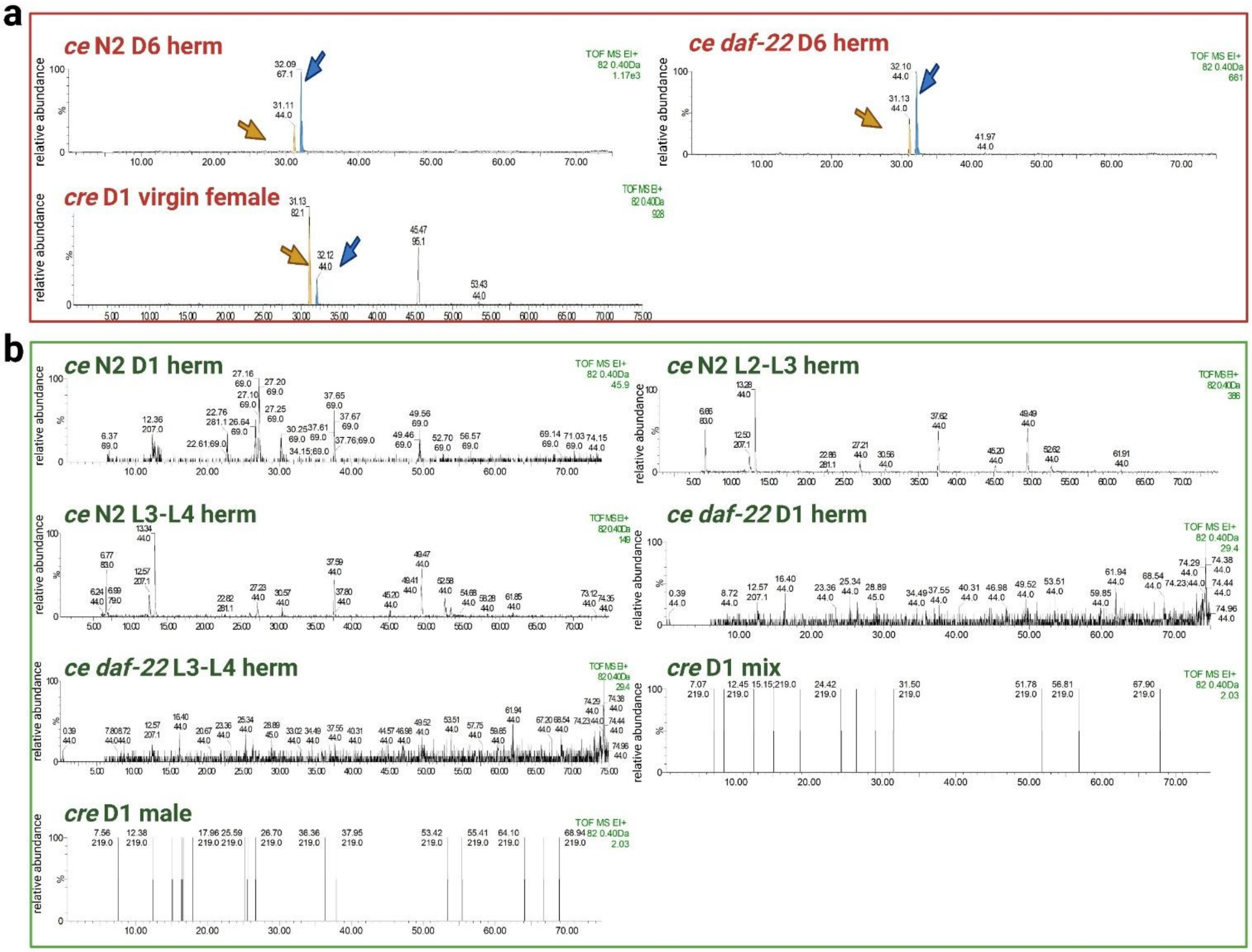

Fig. S2. Full GC-MS EIC for *m/z* 82.0 of pheromone-positive and pheromone-negative samples. *Peaks 1/2* were exclusively observed in pheromone-positive samples. (a) Representative EIC (0–75 min) of pheromone-positive samples, including 6-day-old self-sperm-depleted *C. elegans* N2 (wild-type) and *daf-22* mutant hermaphrodites, and 1-day-old unmated *C. remanei* females. *Peaks 1* (yellow arrows, t_R=31.1min) and *peak 2* (blue arrows, t_R=32.1min) are characteristic of pheromone presence. (b) Representative GC profile (0–75 min) of a pheromone-negative control lacking both peaks.

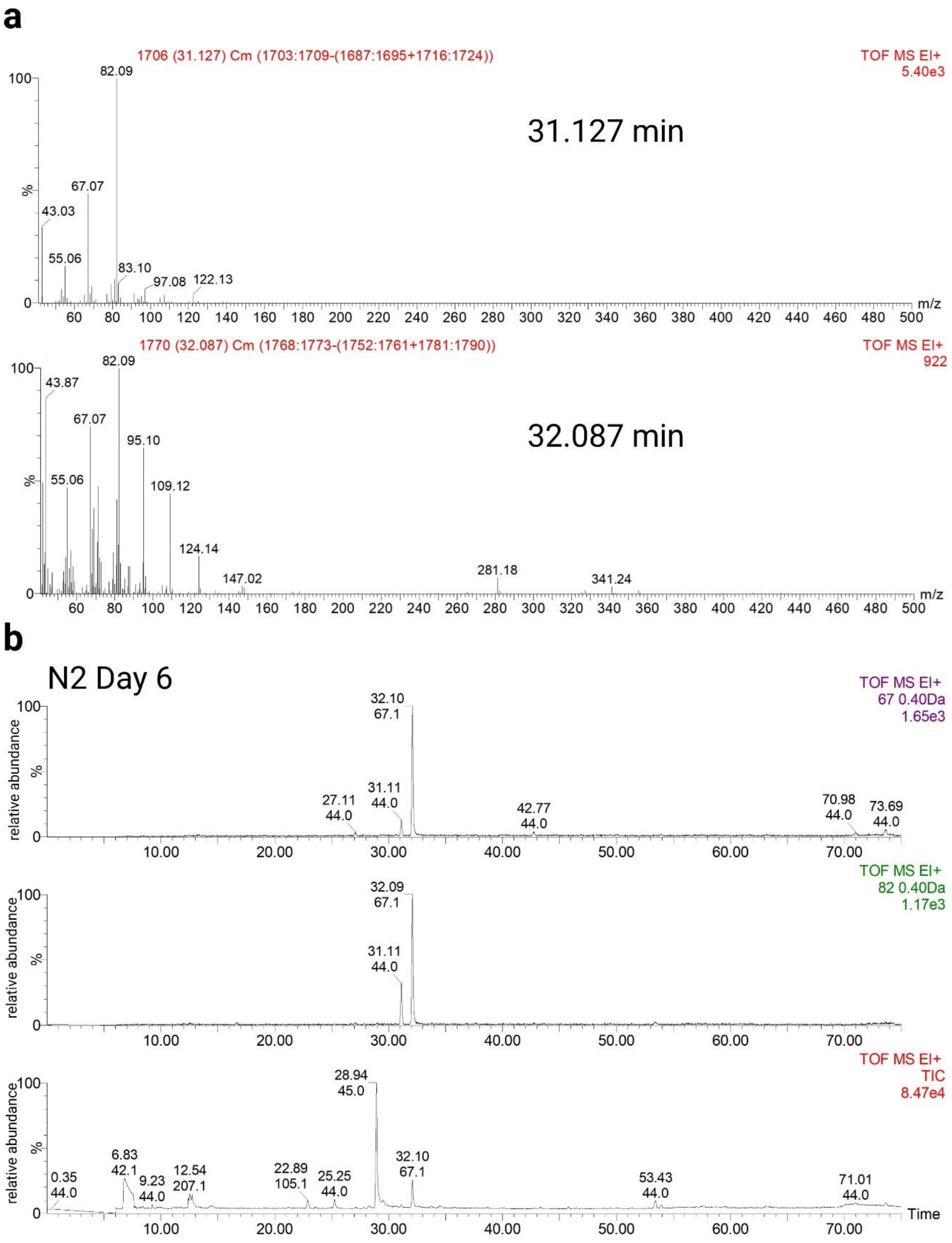

Fig. S3. (a) Full MS (0-500 m/z) of *Peak 1* (t_R=31.1 min) and *Peak 2* (t_R=32.1 min) detected in pheromone-positive samples. Retention times and mass spectra were conserved across samples. (b) GC profiles (0–75 min) of VSPs positive data: 6-day-old N2 hermaphrodites, showing EICs for m/z 67.0 and m/z 82.0, alongside the total ion chromatogram (TIC).

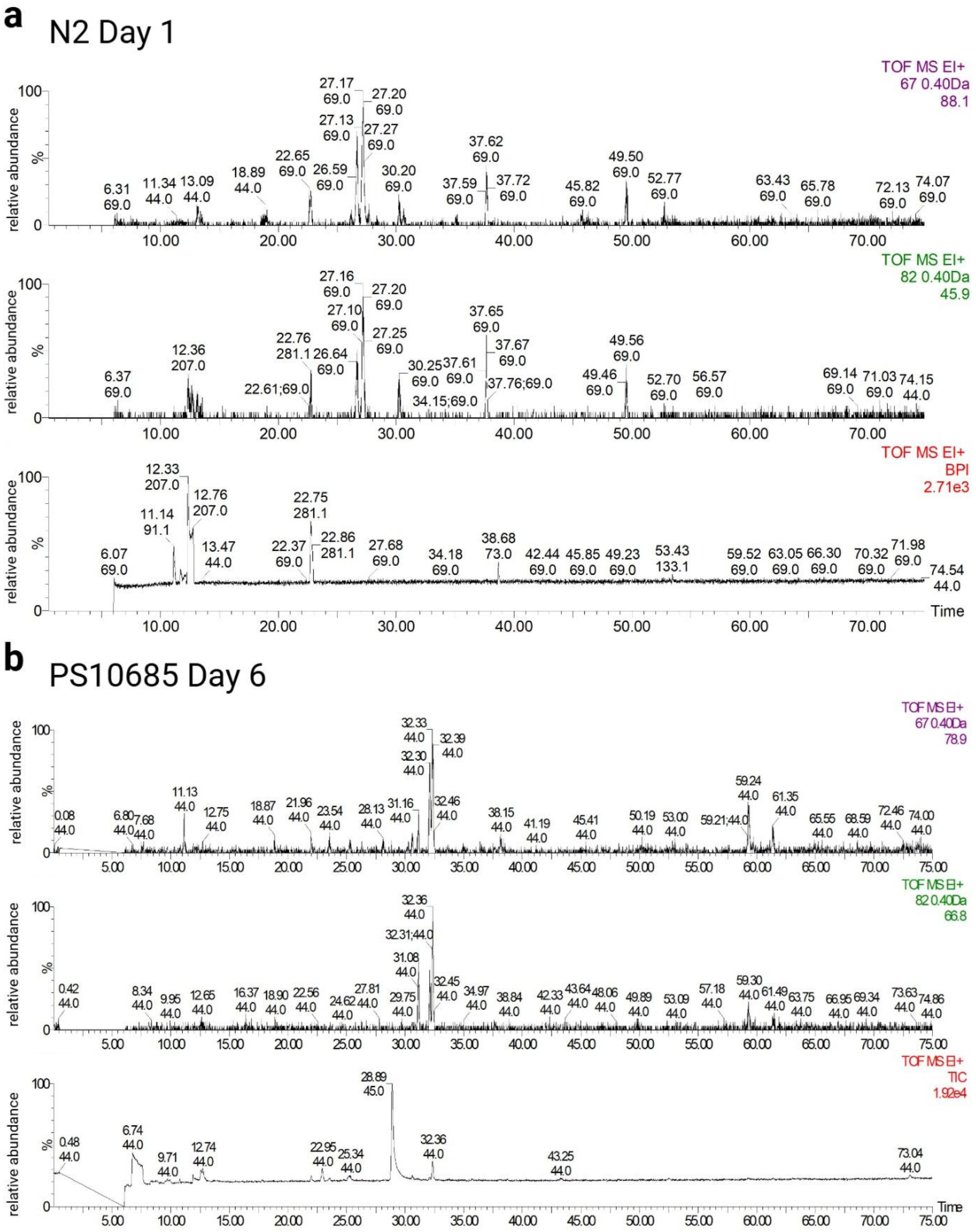

Fig. S4. GC profiles (0–75 min) of VSPs negative data: 1-day-old N2 hermaphrodites and 6-day-old *oac-16 oac-13* double mutant hermaphrodites showing EICs for m/z 67.0 and m/z 82.0, alongside TIC.

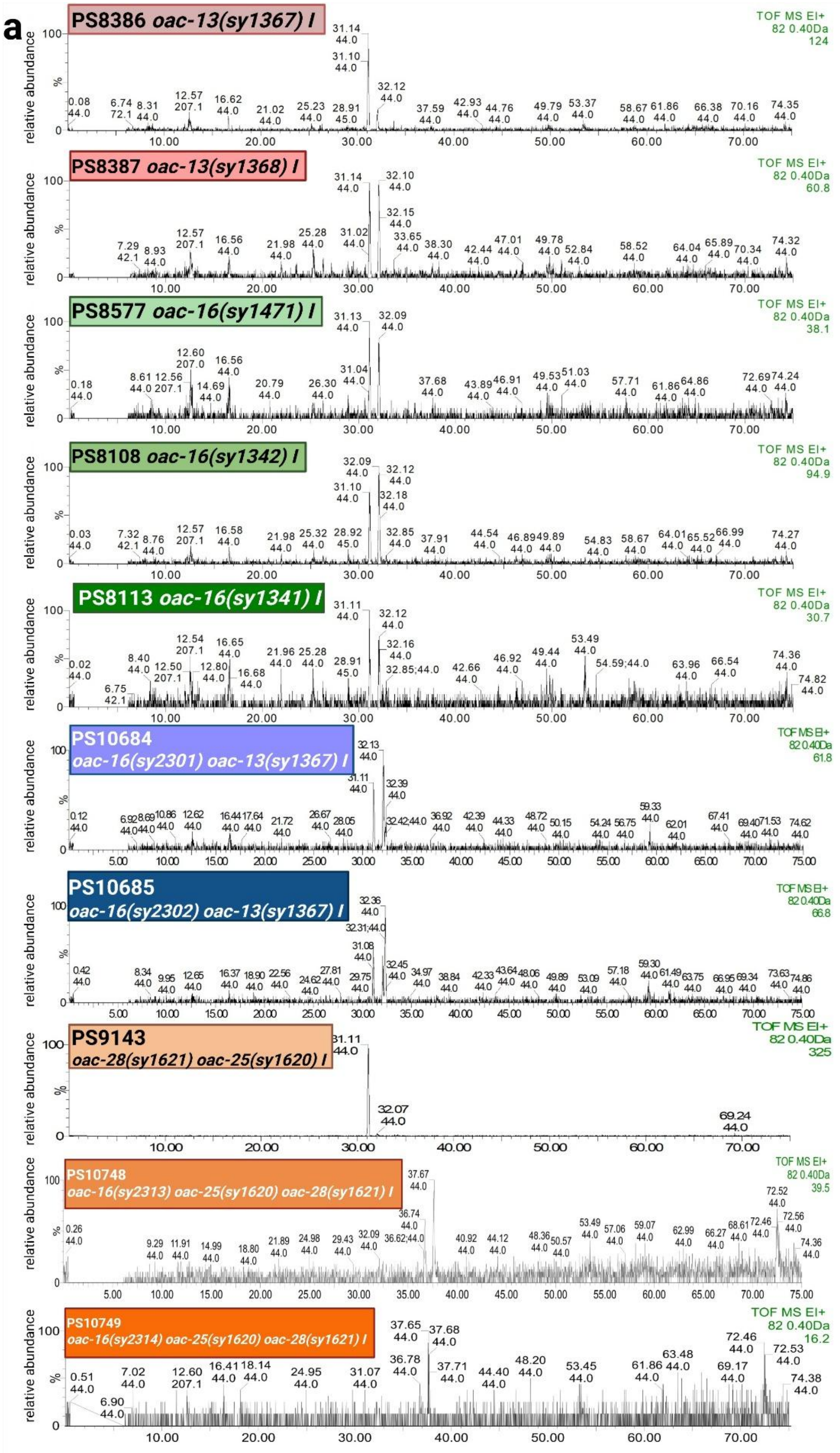
a
PS8386 oac-13(sy1367) I
PS8387 oac-13(sy1368) I
PS8577 oac-16(sy1471) I
PS8108 oac-16(sy1342) I
PS8113 oac-16(sy1341) I
PS10684
oac-16(sy2301) oac-13(sy1367) I
PS10685
oac-16(sy2302) oac-13(sy1367) I
PS9143
oac-28(sy1621) oac-25(sy1620) I
PS10748
oac-16(sy2313) oac-25(sy1620) oac-28(sy1621) I
PS10749
oac-16(sy2314) oac-25(sy1620) oac-28(sy1621) I
relative abundance
TOF MS EI+
82 0.40Da

Fig. S5. Full GC profiles (0–75 min; m/z 82.0 ion) of *oac-13, oac-16, oac-16 oac-13* double mutant, and *oac-28 oac-25* double mutant. *Peaks 1/2* (t_R=31.1 min and 32.1 min), corresponding to pheromone components identified in wild type samples (See Figure 3 and S2), were abolished or markedly reduced in all mutant strains. *oac-13, oac-16* single mutant, and *oac-16 oac-13* double mutant reduced abundances of both peaks similarly. *oac-28 oac-25* double mutant abolished *Peak 2* entirely, while *Peak 1* remained unaffected. (n = 5–8 biological replicates)

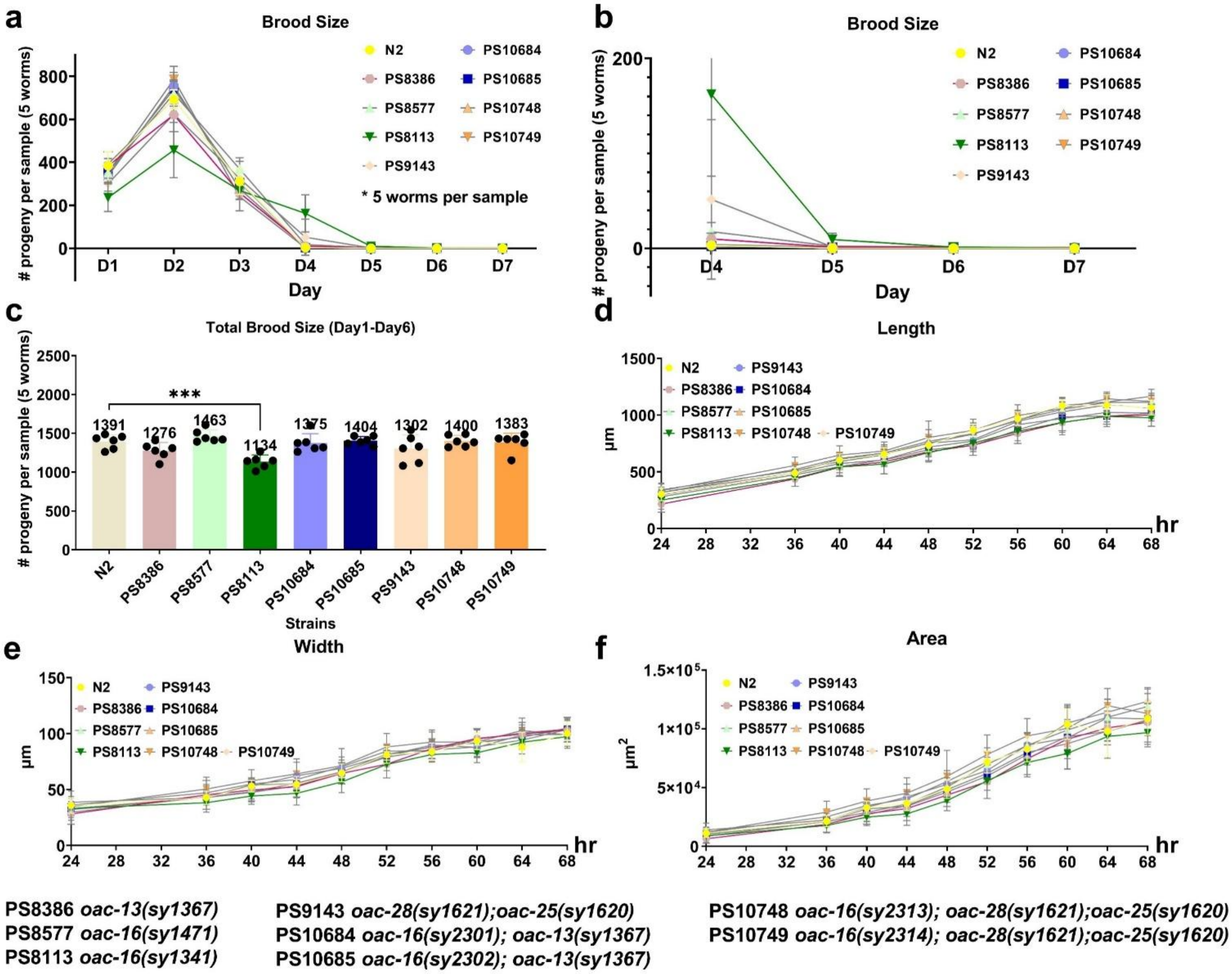

Fig. S6. Brood size and body growth of *oac* mutants. (a) Daily brood size from Day 1–Day 7 for single mutants (*oac-13, oac-16*), double mutants (*oac-25; oac-28, oac-13; oac-16*), and the triple mutant (*oac-16; oac-25; oac-28*), with N2 as wild type control. Counts are reported as "# progeny per worm." For each strain, 6 independent samples (5 hermaphrodites per sample) were scored (total 30 worms/strain). (b) Magnified view of the late reproductive period (Day 4–Day 7) from panel a. (c) Total brood size per 5-worm sample summed across Day 1–Day 7; black dots indicate individual samples and bars show group means. (d–f) Somatic growth over time for the same strains: (d) body length (μm), (e) body width (μm), and (f) body area (μm²). X-axis indicates time (hours) post-egg deposition. Data in all plots are mean ± s.d.

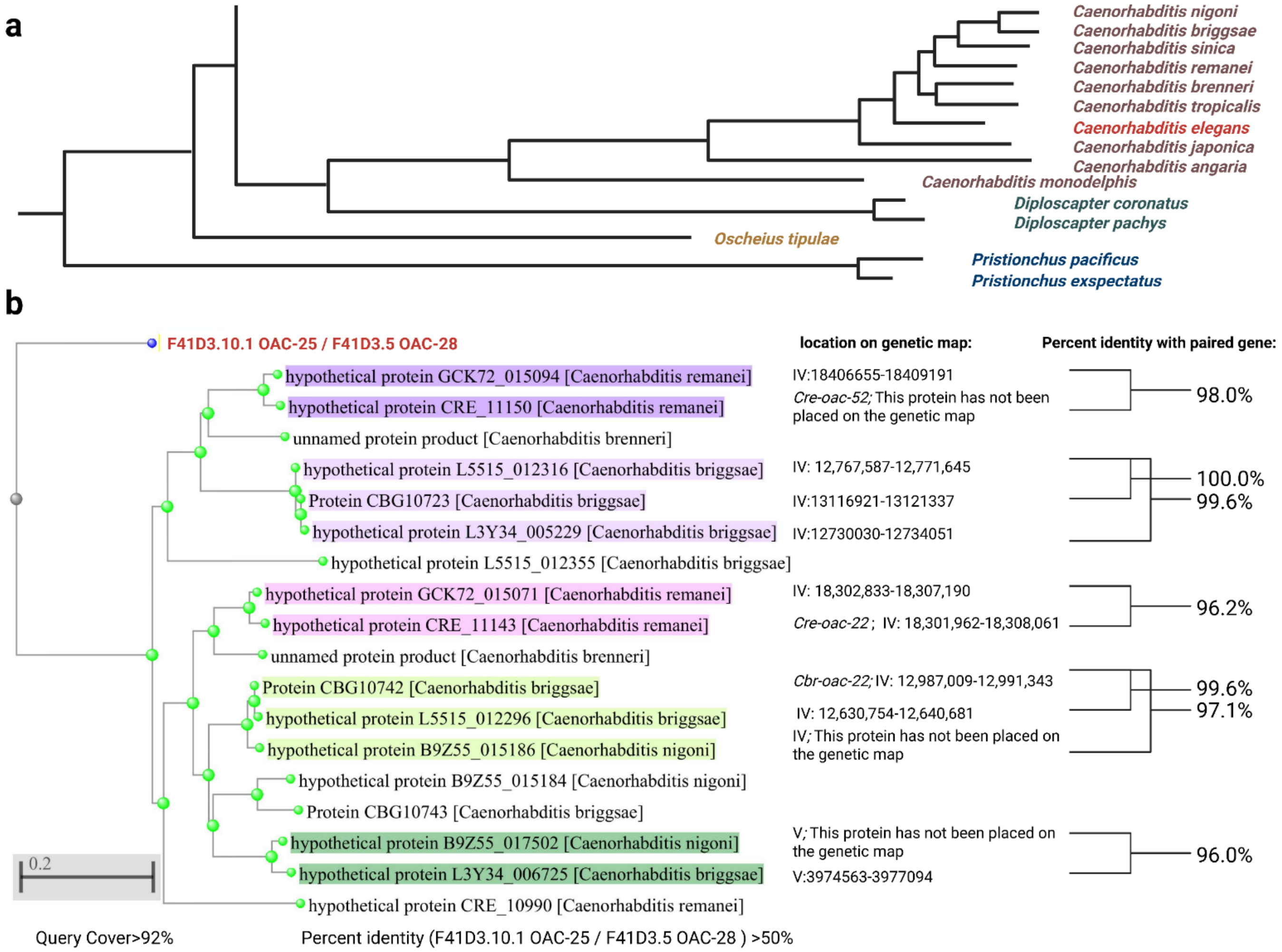

Fig. S7. Lineage-specific gene duplication of OAC-25/OAC-28 homologs occurred within the *Caenorhabditis* lineage prior to and following the divergence of *C. elegans*. (a) Phylogenomic map of *Caenorhabditis* Species, *Diploscapter* Species, *Pristionchus* Species, and *Oscheius tipulae*. Figure modified from (70-72) (b) BLAST analysis of OAC-25/OAC-28 protein sequences in nematode taxa diverging prior to, and subsequent to, the emergence of *C. elegans* shows no identical proteins were detected, with maximum sequence identities of 55.39% (*C. remanei* hypothetical protein GCK72_015070) and 54% (*C. briggsae* CBG10742 and L5515_012296). Among 16 proteins with >92% query coverage and >50% identity to OAC-25/OAC-28, there are two paralog pairs in *C. remanei* and one pair plus three near-identical paralogs in *C. briggsae.* Paralogous protein pairs are highlighted with corresponding colors, while genomic locations and percentage identity values relative to their paralogous counterparts are annotated in the right panel. This analysis and accompanying figures were produced using the NCBI BLASTP tool.

Table S1. 95 alleles representing 56 *oac* genes were made using either a deletion or knock-in CRISPR/Cas9 method.

Genotype	Strain	Description
oac-1(sy1399)	PS8547	605 bp Deletion
oac-2(sy1218)	PS8201	Triple-Stop Knock-In
oac-2(sy1219)	PS8202	Triple-Stop Knock-In
oac-3(sy1329)	PS7536	387 bp Deletion
oac-3(sy1053)	PS7577	1,428 bp Deletion
oac-4(sy1400)	PS8548	5 bp Deletion
oac-5(sy1333)	PS7556	854 bp Deletion
oac-5(sy1334)	PS7791	1,231 bp Deletion
oac-6(sy1326)	PS7466	1,899 bp Deletion
oac-7(sy1327)	PS7490	546 bp Deletion
oac-8(sy1284)	PS8365	Triple-Stop Knock-In
oac-8(sy1285)	PS8366	Triple-Stop Knock-In
oac-9(sy1324)	PS7592	1,129 bp Deletion
oac-10(sy1339)	PS7908	875 bp Deletion
oac-10(sy1340)	PS8110	942 bp Deletion
oac-11(sy1407)	PS8310	1438 bp Deletion
oac-12(sy1378)	PS8439	Triple-Stop Knock-In
oac-12(sy1379)	PS8440	Triple-Stop Knock-In
oac-13(sy1367)	PS8386	708 bp Deletion
oac-13(sy1368)	PS8387	481 bp Deletion
oac-14(sy1343)	PS7593	544 bp Deletion
oac-14(sy1344)	PS7696	868 bp Deletion
oac-14(sy1345)	PS7697	404 bp Deletion
oac-15(sy1369)	PS8388	579 bp Deletion
oac-15(sy1370)	PS8389	835 bp Deletion
oac-16(sy1341)	PS8113	1,255 bp Deletion
oac-16(sy1342)	PS8108	746 bp Deletion
oac-16(sy1471)	PS8577	Triple-Stop Knock-In
oac-17(sy1405)	PS8299	419 bp Deletion
oac-17(sy1406)	PS8300	1,056 bp Deletion
oac-19(sy1741)	PS9361	Triple-Stop Knock-In
oac-20(sy1346)	PS7950	757 bp Deletion
oac-20(sy1347)	PS7956	561 bp Deletion
oac-20(sy1348)	PS8109	988 bp Deletion
oac-21(sy1566)	PS8897	Triple-Stop Knock-In
oac-22(sy1286)	PS8367	Triple-Stop Knock-In
oac-22(sy1287)	PS8368	Triple-Stop Knock-In
oac-23(sy1384)	PS8517	Triple-Stop Knock-In
oac-23(sy1385)	PS8518	Triple-Stop Knock-In
oac-24(sy1246)	PS8250	Triple-Stop Knock-In
oac-24(sy1247)	PS8251	Triple-Stop Knock-In
oac-25(sy1620) ***oac-28(sy1621)***	PS9143	8 bp insert; 8 bp insert
oac-26(sy1386)	PS8519	Triple-Stop Knock-In
oac-27(sy1475)	PS8714	Triple-Stop Knock-In
oac-27(sy1476)	PS8715	Triple-Stop Knock-In
oac-28(sy1734)	PS9351	8 bp insert
oac-29(sy1335)	PS7787	896 bp Deletion
oac-30(sy1577)	PS8934	Triple-Stop Knock-In
oac-30(sy1578)	PS8935	Triple-Stop Knock-In

oac-31(sy1328)	PS7559	591 bp Deletion
oac-32(sy1330)	PS7558	1,764 bp Deletion
oac-34(sy1290)	PS8394	Triple-Stop Knock-In
oac-34(sy1291)	PS8395	Triple-Stop Knock-In
oac-35(sy1390)	PS8538	Triple-Stop Knock-In
oac-35(sy1391)	PS8539	Triple-Stop Knock-In
oac-36(sy1411)	PS8580	Triple-Stop Knock-In
oac-36(sy1412)	PS8581	Triple-Stop Knock-In
oac-37(sy1401)	PS8549	Triple-Stop Knock-In
oac-38(sy1256)	PS8265	Triple-Stop Knock-In
oac-38(sy1257)	PS8266	Triple-Stop Knock-In
oac-39(sy1321)	PS7428	546 bp Deletion
oac-40(sy1258)	PS8301	Triple-Stop Knock-In
oac-40(sy1259)	PS8302	Triple-Stop Knock-In
oac-41(sy1413)	PS8582	Triple-Stop Knock-In
oac-41(sy1414)	PS8583	Triple-Stop Knock-In
oac-42(sy1433)	PS8632	Triple-Stop Knock-In
oac-42(sy1434)	PS8633	Triple-Stop Knock-In
oac-43(sy1381)	PS8480	Triple-Stop Knock-In
oac-44(sy1431)	PS8630	Triple-Stop Knock-In
oac-44(sy1432)	PS8631	Triple-Stop Knock-In
oac-45(sy1742)	PS9362	Triple-Stop Knock-In
oac-46(sy1747)/ mIn1[dpy-10(e128) mIs14]	PS9372	Triple-Stop Knock-In, balanced
oac-46(sy1747)	PS9373	Triple-Stop Knock-In
oac-48(sy1402)	PS8550	Triple-Stop Knock-In
oac-49(sy1380)	PS8479	Triple-Stop Knock-In
oac-50(sy1336)	PS7716	559 bp Deletion
oac-50(sy1337)	PS7790	927 bp Deletion
oac-50(sy1338)	PS7730	782 bp Deletion
oac-51(sy1358)	PS8482	Triple-Stop Knock-In
oac-51(sy1359)	PS8483	Triple-Stop Knock-In
oac-51(sy1388)	PS8536	Triple-Stop Knock-In
oac-51(sy1389)	PS8537	Triple-Stop Knock-In
oac-52(sy1288)	PS8369	Triple-Stop Knock-In
oac-52(sy1289)	PS8370	Triple-Stop Knock-In
oac-53(sy1331)	PS7652	483 bp Deletion
oac-53(sy1332)	PS7687	592 bp Deletion
oac-54(sy1322)	PS7465	1,138 bp Deletion
oac-55(sy1323)	PS7717	1,030 bp Deletion
oac-56(sy1372)	PS8527	Triple-Stop Knock-In
oac-56(sy1373)	PS8528	Triple-Stop Knock-In
oac-57(sy1374)	PS8529	Triple-Stop Knock-In
oac-57(sy1375)	PS8530	Triple-Stop Knock-In
oac-58(sy1376)	PS8531	Triple-Stop Knock-In
oac-58(sy1377)	PS8532	Triple-Stop Knock-In
oac-59(sy1260)	PS8303	Triple-Stop Knock-In
oac-59(sy1261)	PS8304	Triple-Stop Knock-In

Table S2. List of all *oac* mutant strains made by deletion method.

Gene	Strain	Left Flanking Sequence	Right Flank Sequence	Forward Primer	Reverse Primer
oac-1(sy1399)	PS8547	GGTCGGCAGC ATTAGAGACT	GCCAGTTTTTT TTGTTTTCA	GTCGGGTGCT TAAGAGAACT	AAAGGTGATG AGCCACTACA
oac-3(sy1329)	PS7536	GACGAGAAAT AATGGCGTAA	ACTCCGGAAT GCACAAAAGA	TCAACATGGC ATGCGTTTCC	GTCCTGCAAT TGGAGTCCAA
oac-3(sy1053)	PS7577	GACTAACACG TAAAGCCGTT	TCAGTGGACA CATTTTTTCT	TCAACATGGC ATGCGTTTCC	GTCCTGCAAT TGGAGTCCAA
oac-4(sy1400)	PS8548	CTTTTAAAGAT TCTTCGTCC	GGATTCCTCA TGTGCATGCT	CGCTAAATAC GGCCCGCTTT	ACTCGCCTCG TAATCACAGT
oac-5(sy1333)	PS7556	TTTTCTGCGTT ACTTTGTTA	ACAAGAGGAT CAGAAGAGGA	AACTCCAGCC GGACGCGTTT	TATGCATATAG CTGCCAGCG
oac-5(sy1334)	PS7791	TTCTGCGTTAC TTTGTTAGC	ACTCAAAGTG TCACAAATCA	AACTCCAGCC GGACGCGTTT	TATGCATATAG CTGCCAGCG
oac-6(sy1326)	PS7466	TACCAACTACA TTTTTCTTC	TTTTCAGTATC TTCTCCCGT	GTGGCTGAGT TATCTGCAGA	AGGCCAGACG GGAGAAGTTG
oac-7(sy1327)	PS7490	GCATTTGGTA AACTCCCCTC	CTGGATTTATA AAATGCTTG	GGAAGCTGAT AGCTGGGAGG	CACAAGCCAT CCAATCACCG
oac-9(sy1324)	PS7592	TGAAACATGTA AAGAACTAT	CTTTGTACGTT GTATCAGCT	CCTCATCAGC CTTCGTCAGC	ATGGGACACA CGTTGATGGG
oac-10(sy1339)	PS7908	TGTGTTTTTTC AGTGTATTT	CCTACGAGAA ATGGTACCTA	GAACATGGAT TCCGCGTGGA	GATCCTCATT GGCAATGGTC
oac-10(sy1340)	PS8110	AACGAGATGG AAGAACAAAT	TTTCCTTTATT TCCGTTAAA	GAACATGGAT TCCGCGTGGA	GATCCTCATT GGCAATGGTC
oac-11(sy1407)	PS8310	AATAAAGGCC GTCTGATAAA	CGACTTTCAG GCTGTGAAGC	CACTTTATCTG CTTGTCATC	CCAAGGCGTT CTGGAATAAT
oac-13(sy1367)	PS8386	AATAGGGTTTA ACGCTGTGT	TTCTCGTTAAG CTAGTTTTA	GGCACTTTGA AGCCGTTCCT	AGTAGATAGG CCAGTGGAGC
oac-13(sy1368)	PS8387	GTTACGGCTA AGCGATCGGA	TGGACATTTTC ACCCATACA	GGCACTTTGA AGCCGTTCCT	AGTAGATAGG CCAGTGGAGC
oac-14(sy1343)	PS7593	TGAAAGCAAA CTTGTACGCT	GTTTTTTTGAA AATTCTTCT	TTGAGCACTT CAGGTGCAGT	AACATTCCCC ATCCAAGACG
oac-14(sy1344)	PS7696	GAAAGCAAAC TTGTACGCTA	TGACCTGGAT CATGTTTTAC	TTGAGCACTT CAGGTGCAGT	AACATTCCCC ATCCAAGACG
oac-14(sy1345)	PS7697	ACTGAAAGAG CTCAAAATTT	GGGGTTTTTC GTAAGTGCTT	TTGAGCACTT CAGGTGCAGT	AACATTCCCC ATCCAAGACG
oac-15(sy1369)	PS8388	GAAATTGGAA TTTAAGTGCG	TTTCCTAATAA CCCAGTTGG	GTAGTGGGTG ACCTGTCGGC	TTGAACTGTG CCCGGATGTG
oac-15(sy1370)	PS8389	GAAATTGGAA TTTAAGTGCG	CTGTGCTCTTT CTCTGTATT	GTAGTGGGTG ACCTGTCGGC	TTGAACTGTG CCCGGATGTG
oac-16(sy1341)	PS8113	AGCTAAACCG AAGCGTCTAG	TATCTCATATT CCCTATATC	CTGCCTCGGT TGCTTCCAAG	GATAGTCCGG CTTTTCCGCG
oac-16(sy1342)	PS8108	AGCAGCTAAA CCGAAGCGTC	AGTTGACTTCT TTGGAAGCT	CTGCCTCGGT TGCTTCCAAG	GATAGTCCGG CTTTTCCGCG
oac-17(sy1405)	PS8299	AGTAAGTTCC ACTGAGTCTA	GCTCGCCATC GCCGTGAACA	CTAGATGCCA GCTTGTTTTG	AGATACTCTTT GTCTTGGCG
oac-17(sy1406)	PS8300	TCAGTAAGTTC CACTGAGTC	AATTTCTATCC AATAGAGTT	CTAGATGCCA GCTTGTTTTG	AGATACTCTTT GTCTTGGCG
oac-20(sy1346)	PS7950	AACAATGACA CTGAAACATG	TTTTTAAAGTT TGAAATAAT	GGGAATTACT TCGCAAACGC	AACGAACCCA AGGCTTTTGG
oac-20(sy1347)	PS7956	TCAGACCTGC CGGCGTGTTT	CAAGTTATCC CGTTGAGAAG	GGGAATTACT TCGCAAACGC	AACGAACCCA AGGCTTTTGG

oac-20(sy1348)	PS8109	CACTGAAACA TGGTTGCACT	ACATGATTTTT CTCGGTTTC	GGGAATTACT TCGCAAACGC	AACGAACCCA AGGCTTTTGG
oac-25(sy1620)	PS9143	GGAAGCATCT AAACCAAAGC	GATTGGATTT GCAAGGGCTC	CGTTGCCTCG TAGCTGATTT	CTCGCTTCAG TAGCATGCAC
oac-28(sy1621)	PS9143	GGAAGCATCT AAACCAAAGC	GATTGGATTT GCAAGGGCTC	AACAATACCTC CAGCTTGCA	CTCGCTTCAG TAGCATGCAC
oac-28(sy1734)	PS9351	GGAAGCATCT AAACCAAAGC	GATTGGATTT GCAAGGGCTC	AACAATACCTC CAGCTTGCA	CTCGCTTCAG TAGCATGCAC
oac-29(sy1335)	PS7787	ATTTCCCAAAA TTTTCAGCA	TTATTTTCAAG AATTTATAT	TGGTACTTTTG GGCTGAGTC	TCGGATCCAT TCGTGAGAGC
oac-31(sy1328)	PS7559	TTTGGATTACG TCCAAGATG	AAATAGAAACA CCAAAATTT	GCGACCGTAT TGATGGCGAA	TTTGCATGCCT CGACTTGTG
oac-32(sy1330)	PS7558	GCATCCGACT TCTCTCAATG	TGCTGGGTTG TTGGAGCGAA	CTGCTACCTG CTTCTCACAC	GTTCGCTCCA ACAACCCAGC
oac-39(sy1321)	PS7428	CCGTGGTCTT GTAGGAATGA	CATTGTGCCG CGATGCTTAT	CGCTGATTGG TCCTTTGTCG	TGGCTATGTA GTACGCGGGG
oac-50(sy1336)	PS7716	TCATGGGAAA GACTACGCTT	TTTCAGGTCAA CGAATAATA	TGCATTCGCG AGATATCCGG	GAAGGGACGC CAAAACGGAT
oac-50(sy1337)	PS7790	TGGGAAAGAC TACGCTTCAA	GGGAAAACCA TCACTTGATG	TGCATTCGCG AGATATCCGG	GAAGGGACGC CAAAACGGAT
oac-50(sy1338)	PS7730	CATGGGAAAG ACTACGCTTC	GATCCATCCA AAATTTCCTT	TGCATTCGCG AGATATCCGG	GAAGGGACGC CAAAACGGAT
oac-53(sy1331)	PS7652	TTCGTCGCTA CTTCTACACA	GGATTTACGT GAGTTTTTTT	GCAAAGAGGA GGCCCTAAAA	ACAATCGACA CCGCTCCTTA
oac-53(sy1332)	PS7687	ACCTTCGTCG CTACTTCTAC	GTGTGGTGTC GAACCAGACA	GCAAAGAGGA GGCCCTAAAA	ACAATCGACA CCGCTCCTTA
oac-54(sy1322)	PS7465	ACTTTCAATAA TTGTTCATA	AGGACTGTGG TTAATTTATT	TTTGACTCGT GGACAGGAAG	CGTTGAAGCA CCAATGGGAA
oac-55(sy1323)	PS7717	GCGGCACGAT ATGCCCGGCA	CAGGTACCTA CATATTTCCT	TAAGGCTATG GAGCTGCTCG	GAGACAAGGC TTTACTGGGC

Table S3. List of all *oac* mutant strains made by insertion method.

Genotype	Strain	Left Flanking Sequence	Right Flank Sequence	Forward Primer	Reverse Primer
oac-2(sy1218)	PS8201	AGATTTCGAA GAATCCTCCC	GCTGTACTAC TTGACCATCT	TTCATTTGCTGC CTAGCCTG	GAAGCAGTGACG CTATGGAG
oac-2(sy1219)	PS8202	AGATTTCGAA GAATCCTCCC	GCTGTACTAC TTGACCATCT	TTCATTTGCTGC CTAGCCTG	GAAGCAGTGACG CTATGGAG
oac-8(sy1284)	PS8365	TCCCTAAAAC CTTCCCAAAT	GGGTATATTG GAGTAGATAT	GAAACGGGATG ACCTGCAAG	GTAATATCCGCTT GGCTCGAC
oac-8(sy1285)	PS8366	TCCCTAAAAC CTTCCCAAAT	GGGTATATTG GAGTAGATAT	GAAACGGGATG ACCTGCAAG	GTAATATCCGCTT GGCTCGAC
oac-12(sy1378)	PS8439	CCGTCAAAGC GTCAGGATCT	ACAAGGGATC CGCGGAATAG	GGTTGGCTCTCT GCAGGTTG	CCATGTGTGAGT GAACAAGT
oac-12(sy1379)	PS8440	CCGTCAAAGC GTCAGGATCT	ACAAGGGATC CGCGGAATAG	GGTTGGCTCTCT GCAGGTTG	CCATGTGTGAGT GAACAAGT
oac-16(sy1471)	PS8577	AAACCGAAGC GTCTAGATTT	ACAAGGTATC CGTGGTCTCG	GCAAAAGCACT GGGAGTGAT	GATAGTCCGGCT TTTCCGCG
oac-19(sy1741)	PS9361	GCAGTTCTAG GCTTCCACTT	CTACCCTGAC ACCTTCCCAA	GTTTCAGATGG GTTCCTTGC	TGTTATTAAAGTA CATGGTGGCTC
oac-21(sy1566)	PS8897	AACGACTAGA TCTTCAAGGC	ATCCGGGCTC TCGCTATTCT	CGTTTTACTCTT TGATGCCTCCG	TAGAAACCCGGA GAGCACAAAG
oac-22(sy1286)	PS8367	TGTGTCAAAC AGACCAAAAA	CTGTGGCAGA GGACTATTTT	TGCTGAAGCGA GCTGAGAATC	AACGATATTGCC CGCACCTAG
oac-22(sy1287)	PS8368	TGTGTCAAAC AGACCAAAAA	CTGTGGCAGA GGACTATTTT	TGCTGAAGCGA GCTGAGAATC	AACGATATTGCC CGCACCTAG
oac-23(sy1384)	PS8517	CAGTCGACAT CTTTACACAT	ACCTGGTCAC TCTCAGTGGA	TAAGGAGTCTG CTCTTCACG	CAGGTTGCTGAG CACTCGAT
oac-23(sy1385)	PS8518	CAGTCGACAT CTTTACACAT	ACCTGGTCAC TCTCAGTGGA	TAAGGAGTCTG CTCTTCACG	CAGGTTGCTGAG CACTCGAT
oac-24(sy1246)	PS8250	CTTTGTCATTT CCGGATACC	TCATGGCGAA AAATTTAACG	TTGAATTGAAGG CCACCTACG	AGTAACCAAGCA CAACGACG
oac-24(sy1247)	PS8251	CTTTGTCATTT CCGGATACC	TCATGGCGAA AAATTTAACG	TTGAATTGAAGG CCACCTACG	AGTAACCAAGCA CAACGACG
oac-26(sy1386)	PS8519	TGATTGTAACA CGATTACCA	GCACGGTTTC ACATTGCGTC	GACACGAGCCC TGTTTTTCG	GCGGTGCACCTG ATTATAGA
oac-27(sy1475)	PS8714	CCATGACAAG CCAACCTGCA	AAGCGGCTGG ACCTGCAAGG	TAAGGTTTACAT CCCGTGCG	ATCCTCCAATGT CGATGCCC
oac-27(sy1476)	PS8715	CCATGACAAG CCAACCTGCA	AAGCGGCTGG ACCTGCAAGG	TAAGGTTTACAT CCCGTGCG	ATCCTCCAATGT CGATGCCC
oac-30(sy1577)	PS8934	GAAATTTTCAG ATTCCGCCG	AATCCTGCCA CTCTACTATC	AGAAACCTTACC AAGTCGAAGC	GAAACAGGGAGG CAATGGAG
oac-30(sy1578)	PS8935	GAAATTTTCAG ATTCCGCCG	AATCCTGCCA CTCTACTATC	AGAAACCTTACC AAGTCGAAGC	GAAACAGGGAGG CAATGGAG
oac-34(sy1290)	PS8394	CGGTTACCTG ATGGCCCGTA	ACCTGACACA CATGAAAATC	GCCGCTTTGTC GGGTTTATC	GCAGACGTGAAC TGCAATGG
oac-34(sy1291)	PS8395	CGGTTACCTG ATGGCCCGTA	ACCTGACACA CATGAAAATC	GCCGCTTTGTC GGGTTTATC	GCAGACGTGAAC TGCAATGG
oac-35(sy1390)	PS8538	GTTGCTCAAG CGTGCCGAGA	CCCACCCATT TTTCACGTTG	GTGGCTTGGCT ATACTTGTTG	TGTAGAGGCAAA TGGCAGAG
oac-35(sy1391)	PS8539	GTTGCTCAAG CGTGCCGAGA	CCCACCCATT TTTCACGTTG	GTGGCTTGGCT ATACTTGTTG	TGTAGAGGCAAA TGGCAGAG

oac-36(sy1411)	PS8580	CAAGCGTGCC GAGACCAAGC	CATTTTTCACA GTGGTATGC	GTGGCTTGGCT ATACTTGTTGTA C	GTTAGCCAGAAG TGATCCCAAG
oac-36(sy1412)	PS8581	CAAGCGTGCC GAGACCAAGC	CATTTTTCACA GTGGTATGC	GTGGCTTGGCT ATACTTGTTGTA C	GTTAGCCAGAAG TGATCCCAAG
oac-37(sy1401)	PS8549	TGATATCGGG GTACCTGATG	GCTCGGAACT TGACACATTC	CTTCTGTGCCGA CGCATTGT	GTTCCATCTGCC AGGTACTG
oac-38(sy1256)	PS8265	TTCCTGCCAG ATGTATTTCC	TAATGGATACT TAGGAGTTG	CTCAAGGAGTTC GAGGCTTGG	GTCAGCAGGGCA GTATCTGG
oac-38(sy1257)	PS8266	TTCCTGCCAG ATGTATTTCC	TAATGGATACT TAGGAGTTG	CTCAAGGAGTTC GAGGCTTGG	GTCAGCAGGGCA GTATCTGG
oac-40(sy1258)	PS8301	TGGGAAACTA ATAACCGGTA	TTCGTTGGCTT CATTATTTT	GTCGAGTTTGA GATCGGTTCAA G	TGAAATAATCTGC CTGGTCGTG
oac-40(sy1259)	PS8302	TGGGAAACTA ATAACCGGTA	TTCGTTGGCTT CATTATTTT	GTCGAGTTTGA GATCGGTTCAA G	TGAAATAATCTGC CTGGTCGTG
oac-41(sy1413)	PS8582	TACACGTTTTT CTACCGGAT	TTCCTGTGGC AAAATAATAA	ACAATGCTTACG TGGTTTGG	TCCGACTGATCG TGAATAACC
oac-41(sy1414)	PS8583	TACACGTTTTT CTACCGGAT	TTCCTGTGGC AAAATAATAA	ACAATGCTTACG TGGTTTGG	TCCGACTGATCG TGAATAACC
oac-42(sy1433)	PS8632	AATTCGGGGT CTCGCCATTG	CTGCAGTGCT TCTTTATCAC	GCTTCAAAGAG GCTTGACTTAC	AGGAGCCGTTTG AATCTTCG
oac-42(sy1434)	PS8633	AATTCGGGGT CTCGCCATTG	CTGCAGTGCT TCTTTATCAC	GCTTCAAAGAG GCTTGACTTAC	AGGAGCCGTTTG AATCTTCG
oac-43(sy1381)	PS8480	GATTTGCAAG CAATCCGAGG	ATTGGCCATT CTATCAGTAC	GTCTCTTTGACG GTCTGTTC	GTTCCCTGACAG CACATCTC
oac-44(sy1431)	PS8630	TTCTACCCAAA CCAGTTTCC	CAATGGATAC CTCGGAGTGG	CCTTCTAAACGG CAGGATTTG	ATCCAGAGAGGA CAAAGAACC
oac-44(sy1432)	PS8631	TTCTACCCAAA CCAGTTTCC	CAATGGATAC CTCGGAGTGG	CCTTCTAAACGG CAGGATTTG	ATCCAGAGAGGA CAAAGAACC
oac-45(sy1742)	PS9362	TCTACCCTAAT CAGTTTCCC	AATGGGTACC TTGGAGTTGA	AGTTTCTTCAAA GCGGCAGG	GTCTTCAAATCC GGTCTTTTTACG
oac-46(sy1747)/ mln1[dpy-10(e128) mls14]	PS9372	TCCTGTTATTA TCACCGGCT	ATTGCTTCAG CCGGTCTAGA		
oac-46(sy1747)/ mln1[dpy-10(e128) mls14]	PS9373	TCCTGTTATTA TCACCGGCT	ATTGCTTCAG CCGGTCTAGA		
oac-48(sy1402)	PS8550	TTCGAGTCAA ATAGGCCGCA	TACTGGAGAG GAAAATTATT	CGCGGATTCGC AATAATTTC	GATGCGATTGAG AGCTGGAA
oac-49(sy1380)	PS8479	TCCCGACTCA GTTTCCAAAT	GGCTATTTGG GAGTGGATCA	GGTACCCCTTTA AATTCCCC	CCCACCACGACA ATAACCGA
oac-51(sy1358)	PS8482	TCTACCCAGA AGTATTTCCA	AATGGGTATC TTGGAGTTGA	CATTGTGCTTGG TGATTCTGG	GGGACTAGAGGT CTTTGACATTC
oac-51(sy1359)	PS8483	TCTACCCAGA AGTATTTCCA	AATGGGTATC TTGGAGTTGA	CATTGTGCTTGG TGATTCTGG	GGGACTAGAGGT CTTTGACATTC
oac-51(sy1388)	PS8536	GCATGGTAGT TTACACCGCT	CTGTGGAACA TTGAAGATCA	CACTACCCTCG CCCGTTATC	ATTCAGTCGGTT GTTGTCATCG
oac-51(sy1389)	PS8537	GCATGGTAGT TTACACCGCT	CTGTGGAACA TTGAAGATCA	CACTACCCTCG CCCGTTATC	ATTCAGTCGGTT GTTGTCATCG
oac-52(sy1288)	PS8369	GGTCTTGCTA TTACAGTTGT	ACTAGGTTTTC ATTTCTATC	GATCTAGTGAAG CAGAAACCGTC	TGACTAAAGAGC ACGTGGGC

oac-52(sy1289)	PS8370	GGTCTTGCTA TTACAGTTGT	ACTAGGTTTTC ATTTCTATC	GATCTAGTGAAG CAGAAACCGTC	TGACTAAAGAGC ACGTGGGC
oac-56(sy1372)	PS8527	CACTTGAACC CTAACCTATT	TGTTAATGGAT TTCTCGGTG	AATGCGAGAAG ACATCCAATGC	CCATGAACAGCG ATGCCAAC
oac-56(sy1373)	PS8528	CACTTGAACC CTAACCTATT	TGTTAATGGAT TTCTCGGTG	AATGCGAGAAG ACATCCAATGC	CCATGAACAGCG ATGCCAAC
oac-57(sy1374)	PS8529	TCAATTTTATT CTTCCATCT	AAAGTCTCTTT TAACAGTTT	ACCTGTTCACTC ATACATGGTC	CTTTCACATTCGC CATGCTC
oac-57(sy1375)	PS8530	TCAATTTTATT CTTCCATCT	AAAGTCTCTTT TAACAGTTT	ACCTGTTCACTC ATACATGGTC	CTTTCACATTCGC CATGCTC
oac-58(sy1376)	PS8531	TTCTATTCAAT TCTCCCACT	TGAAGTGGCT TTTAACAGTT	CCCGTACACGG TTTAATTCTCTC	TTCGTCTTCCACT TCATCTTCC
oac-58(sy1377)	PS8532	TTCTATTCAAT TCTCCCACT	TGAAGTGGCT TTTAACAGTT	CCCGTACACGG TTTAATTCTCTC	TTCGTCTTCCACT TCATCTTCC
oac-59(sy1260)	PS8303	CGGCTGGACC TTCAAGGCAT	TAGAGGGTTG GCAATTCTAT	CTCGAACAATGC CATCCCAG	AGGCGAGTAAAG TAAACCCAAG
oac-59(sy1261)	PS8304	CGGCTGGACC TTCAAGGCAT	TAGAGGGTTG GCAATTCTAT	CTCGAACAATGC CATCCCAG	AGGCGAGTAAAG TAAACCCAAG

Table S4. List of all oac mutant strains used in this paper.

Genotype	Strain	Source
***C. elegans* Wildtype**	N2	Brenner, 1974; *Caenorhabditis* Genomics Center (CGC)
oac-1(sy1399)	PS8547	This work
oac-2(sy1218)	PS8201	This work
oac-2(sy1219)	PS8202	This work
oac-3(sy1329)	PS7536	This work
oac-3(sy1053)	PS7577	This work
oac-4(sy1400)	PS8548	This work
oac-5(sy1333)	PS7556	This work
oac-5(sy1334)	PS7791	This work
oac-6(sy1326)	PS7466	This work
oac-7(sy1327)	PS7490	This work
oac-8(sy1284)	PS8365	This work
oac-8(sy1285)	PS8366	This work
oac-9(sy1324)	PS7592	This work
oac-10(sy1339)	PS7908	This work
oac-10(sy1340)	PS8110	This work
oac-11(sy1407)	PS8310	This work
oac-12(sy1378)	PS8439	This work
oac-12(sy1379)	PS8440	This work
oac-13(sy1367)	PS8386	This work
oac-13(sy1368)	PS8387	This work
oac-14(sy1343)	PS7593	This work
oac-14(sy1344)	PS7696	This work
oac-14(sy1345)	PS7697	This work
oac-15(sy1369)	PS8388	This work
oac-15(sy1370)	PS8389	This work
oac-16(sy1341)	PS8113	This work
oac-16(sy1342)	PS8108	This work
oac-16(sy1471)	PS8577	This work
oac-17(sy1405)	PS8299	This work
oac-17(sy1406)	PS8300	This work
oac-19(sy1741)	PS9361	This work
oac-20(sy1346)	PS7950	This work
oac-20(sy1347)	PS7956	This work
oac-20(sy1348)	PS8109	This work
oac-21(sy1566)	PS8897	This work
oac-22(sy1286)	PS8367	This work
oac-22(sy1287)	PS8368	This work
oac-23(sy1384)	PS8517	This work
oac-23(sy1385)	PS8518	This work
oac-24(sy1246)	PS8250	This work
oac-24(sy1247)	PS8251	This work
oac-25(sy1620);oac-28(sy1621)	PS9143	This work
oac-26(sy1386)	PS8519	This work
oac-27(sy1475)	PS8714	This work
oac-27(sy1476)	PS8715	This work
oac-28(sy1734)	PS9351	This work
oac-29(sy1335)	PS7787	This work
oac-30(sy1577)	PS8934	This work
oac-30(sy1578)	PS8935	This work
oac-31(sy1328)	PS7559	This work

oac-32(sy1330)	PS7558	This work
oac-34(sy1290)	PS8394	This work
oac-34(sy1291)	PS8395	This work
oac-35(sy1390)	PS8538	This work
oac-35(sy1391)	PS8539	This work
oac-36(sy1411)	PS8580	This work
oac-36(sy1412)	PS8581	This work
oac-37(sy1401)	PS8549	This work
oac-38(sy1256)	PS8265	This work
oac-38(sy1257)	PS8266	This work
oac-39(sy1321)	PS7428	This work
oac-40(sy1258)	PS8301	This work
oac-40(sy1259)	PS8302	This work
oac-41(sy1413)	PS8582	This work
oac-41(sy1414)	PS8583	This work
oac-42(sy1433)	PS8632	This work
oac-42(sy1434)	PS8633	This work
oac-43(sy1381)	PS8480	This work
oac-44(sy1431)	PS8630	This work
oac-44(sy1432)	PS8631	This work
oac-45(sy1742)	PS9362	This work
oac-46(sy1747)/ mIn1[dpy-10(e128) mIs14]	PS9372	This work
oac-46(sy1747)/ mIn1[dpy-10(e128) mIs14]	PS9373	This work
oac-48(sy1402)	PS8550	This work
oac-49(sy1380)	PS8479	This work
oac-50(sy1336)	PS7716	This work
oac-50(sy1337)	PS7790	This work
oac-50(sy1338)	PS7730	This work
oac-51(sy1358)	PS8482	This work
oac-51(sy1359)	PS8483	This work
oac-51(sy1388)	PS8536	This work
oac-51(sy1389)	PS8537	This work
oac-52(sy1288)	PS8369	This work
oac-52(sy1289)	PS8370	This work
oac-53(sy1331)	PS7652	This work
oac-53(sy1332)	PS7687	This work
oac-54(sy1322)	PS7465	This work
oac-55(sy1323)	PS7717	This work
oac-56(sy1372)	PS8527	This work
oac-56(sy1373)	PS8528	This work
oac-57(sy1374)	PS8529	This work
oac-57(sy1375)	PS8530	This work
oac-58(sy1376)	PS8531	This work
oac-58(sy1377)	PS8532	This work
oac-59(sy1260)	PS8303	This work
oac-59(sy1261)	PS8304	This work
rhy-1(ok1402)	RB1297	Shen *et al.* 2006; *Caenorhabditis* Genomics Center (CGC)
bus-1(e2678)	CB5609	Gravato-Nobre *et al.* 2005; *Caenorhabditis* Genomics Center (CGC)
ndg-4(sa529)	JT529	Choy and Thomas 1999; *Caenorhabditis* Genomics Center (CGC)

nrf-6(sa525)	JT525	Choy and Thomas 1999; *Caenorhabditis* Genomics Center (CGC)
oac-16(sy2301) oac-13(sy1367)	PS10684	This work
oac-16(sy2302) oac-13(sy1367)	PS10685	This work
oac-16(sy2313) oac-28(sy1621) oac-25(sy1620)	PS10748	This work
oac-16(sy2314) oac-28(sy1621) oac-25(sy1620)	PS10749	This work
